# Increased prevalence of coronary heart disease among current smokers carrying *APOL1* risk variants within the African American population

**DOI:** 10.1016/j.jacl.2025.04.189

**Published:** 2025-04-10

**Authors:** Jelena Mustra Rakic, Clive R. Pullinger, Erin L. Van Blarigan, Irina Movsesyan, Eveline Oestreicher Stock, Mary J. Malloy, John P. Kane

**Affiliations:** Cardiovascular Research Institute, University of California San Francisco, San Francisco, CA; Center for Tobacco Control Research and Education, University of California San Francisco, San Francisco, CA; Cardiovascular Research Institute, University of California San Francisco, San Francisco, CA; Department of Physiological Nursing, University of California San Francisco, San Francisco, CA; Department of Epidemiology and Biostatistics, University of California San Francisco, San Francisco, CA; Cardiovascular Research Institute, University of California San Francisco, San Francisco, CA; Cardiovascular Research Institute, University of California San Francisco, San Francisco, CA; Department of Medicine, University of California San Francisco, San Francisco, CA; Cardiovascular Research Institute, University of California San Francisco, San Francisco, CA; Department of Medicine, University of California San Francisco, San Francisco, CA; Cardiovascular Research Institute, University of California San Francisco, San Francisco, CA; Center for Tobacco Control Research and Education, University of California San Francisco, San Francisco, CA; Department of Medicine, University of California San Francisco, San Francisco, CA; Department of Biochemistry and Biophysics, University of California San Francisco, San Francisco, CA

**Keywords:** African American adults, Cigarette smoking, Apolipoprotein L1, Coronary heart disease, Cross-sectional study

## Abstract

**BACKGROUND:**

The apolipoprotein L1 *(APOL1*) G1 and G2 gene variants, highly prevalent among the African American population (rare in other racial groups), are linked to increased risk of kidney disease, sepsis, and potentially coronary heart disease (CHD). Their role in tobacco-related CHD remains unclear.

**OBJECTIVE:**

To investigate the effect of *APOL1* risk variants on the association between tobacco smoking and prevalent CHD in African American adults.

**METHODS:**

We conducted a cross-sectional study involving 519 African American adults recruited through the University of California San Francisco Lipid Clinic. Using multivariable logistic regression, we assessed the association between tobacco smoking and CHD, overall and with its most severe subtype, myocardial infarction (MI), among all participants and *APOL1* genotype subgroups.

**RESULTS:**

Among participants, 41% were current (14%) or former (27%) smokers, 54% carried *APOL1* risk variants (1 or 2 alleles), and 28% had CHD, including 16% having MI. Current smokers with *APOL1* risk variants had 3.3 times higher odds of CHD compared to nonsmokers (95% CI: 1.6, 6.8), with the strongest effect observed in those with 2 risk alleles (odds ratio [OR]: 7.3, CI: 1.1, 48.6) and a substantial effect in carriers of a single risk allele (OR: 3.2, CI: 1.5, 7.2). Among non-carriers, current smoking was not significantly associated with CHD (OR: 1.3). A similar trend was observed for MI. Former smoking was associated with CHD (OR: 2.0), independent of *APOL1* genotype.

**CONCLUSION:**

African American smokers with *APOL1* G1 and/or G2 risk variants may be at greater risk of CHD; this relationship appears to follow an additive model.

## Introduction

African American individuals bear a high burden of coronary heart disease (CHD), with age-adjusted mortality rates exceeding those of other racial and ethnic groups in the United States.^1–3^ Despite ongoing efforts to reduce cigarette smoking, a well-established risk factor for CHD, African Americans continue to have the lowest quit rates compared to Hispanics/Latinos and non-Hispanic Whites.^4^ Recent findings indicate that smoking disproportionately increases CHD risk in African Americans.^5^ Even after accounting for traditional risk factors like obesity, diabetes, hypertension, and socioeconomic status, African American smokers still face a higher risk of developing tobacco-related CHD compared to White smokers.^5^

The apolipoprotein L1 (APOL1), found in high-density lipoprotein (HDL) and highly expressed in the vasculature, protects against *Trypanosoma brucei*, the parasite responsible for African sleeping sickness.^6–9^ Two rare variants (RVs) exist in the C-terminus: G1 and G2.^6^ G1 consists of 2 mutations (S342G and I384M) in linkage disequilibrium. G2 involves a 2-amino acid deletion (del388N389Y).^6,7,10^ Both provide extended protection against African *Trypanosoma brucei*.^11,12^ Homozygosity or compound heterozygosity is strongly associated with increased risk of kidney disease, sepsis, and potentially cardiovascular disease in the African American population.^6,10,13–17^

Findings on the link between these RVs and CHD are inconclusive.^17,18^ Evidence indicates a strong need to understand the role of other risk factors surrounding *APOL1* RV-associated diseases. The *second hit* hypothesis suggests that the presence of *APOL1* RVs alone, particularly a single risk allele, may be insufficient to trigger disease. Additional factors, such as inflammatory insults, may be required to increase *APOL1* expression to a critical threshold that initiates disease onset.^13,18–20^ *APOL1* RV carriers exhibit endothelial cell dysfunction^13^ and increased risk of thrombotic coronary death, the latter linked to APOL1 protein accumulation in the necrotic core and increased plaque instability.^21^ Smoking elevates systemic inflammation,^22,23,24^ which can increase *APOL1* gene expression.^25^ Tobacco-related inflammation may act as a *second hit* by upregulating *APOL1* gene expression, potentially exacerbating endothelial dysfunction and necrotic core enlargement, ultimately increasing CHD risk in smokers with *APOL1* RV.

Approximately 50% of African American people carry at least 1 *APOL1* risk allele.^6,16,17,19^ These RVs are exceedingly rare in other racial groups. Given this high frequency of RVs and the proinflammatory effects of smoking, we aimed to determine if the presence of these RVs modifies the relationship between tobacco smoking and CHD prevalence in African American adults.

## Materials and methods

### Study design and participants

We conducted a retrospective cross-sectional study using samples and data from participants in the University of California, San Francisco (UCSF) Genomic Resource in Arteriosclerosis (GRA) study, which adhered to the World Medical Association Declaration of Helsinki and was approved by the UCSF Institutional Review Board as part of the UCSF Human Research Protection Program. The GRA includes a large repository of DNA and plasma samples with clinical data, collected over >20 years. Participants were recruited through the UCSF Lipid Clinic, where individuals with dyslipidemia, CHD, diabetes, obesity, other metabolic concerns, or a family history of these conditions were referred. All participants provided written informed consent prior to enrollment. The proportion of African Americans recruited is consistent with their representation in the Bay Area population (approximately 7%^26^). For our study, we included only those who self-identified as African American, were 18 years or older, and had available clinical data and DNA samples. Of 527 identified African American adults, 519 were eligible for analysis (8 were excluded due to missing CHD history). Data are available upon reasonable request.

### Ascertainment of CHD, APOL1 genotype status, and smoking status

Histories of CHD were obtained as before.^27^ Briefly, patients were considered to have CHD if they met any of these criteria: history of myocardial infarction (MI), evidence of coronary disease on angiogram, previous stent placement, angioplasty, or coronary revascularization. Angiographic disease was graded by a “sum score” (sum of percent luminal intrusion of all lesions). A sum score of 60 was the threshold discriminant for diagnosis of CHD.^27^ MI was determined based on specific criteria: elevated troponin levels, electrocardiographic evidence, and/or echocardiographic studies.

Blood was collected after overnight fasting in tubes containing 0.1% ethylenediaminetetraacetic acid and was centrifuged at 1000 *g* for 15 minutes at 4 °C to separate plasma. Plasma was stored at −80 °C. Genomic DNA was extracted using the Wizard purification kit (Qiagen) and *APOL1* genotypes determined by Sanger sequencing as before.^19^ Participants were grouped into *APOL1* RV group (1 or 2 risk alleles: G1/G0, G2/G0, G1/G1, G2/G2, G1/G2) or a reference group (no risk alleles: G0/G0).

Smoking status was obtained from the questionnaire and validated using plasma nicotine and cotinine levels measured at the UCSF Tobacco Biomarkers Core Facility as previously.^19^ This identifies active smoking but lacks sensitivity to detect secondhand smoke exposure (eg, plasma cotinine level greater than 5.92 ng/mL classifies current smokers among African American individuals).^28^ We compared self-reported smoking status with nicotine and cotinine levels (quantitation limits: 1 ng/mL for nicotine, 10 ng/mL for cotinine). Participants with detectable cotinine and/or nicotine were reclassified, where appropriate, as current smokers. Smoking history was assessed by classifying those not identified as current smokers as nonsmokers. Nonsmokers were further categorized as former smokers if they had a history of smoking, or as never smokers if they did not.

### Clinical and lifestyle covariates

Plasma levels of total cholesterol (TC), HDL cholesterol (HDL-C), and triglyceride (TG) were measured as previously.^29^ Hypertension, type 2 diabetes mellitus, and dyslipidemia were defined as before.^19^ Being physically active was defined if participants reported exercising ≥30 minutes (including walking) more than twice per week, and alcohol intake was defined as consuming >2 alcoholic drinks/week. To assess kidney function, we measured plasma creatinine (mg/dL) (QuantiChrom Kit, BioAssay Systems, Hayward, CA). Using creatinine levels, age, and sex (defined as sex assigned at birth), estimated glomerular filtration rate (eGFR) was calculated; eGFR<60 mL/min/1.73 m^2^ indicated mild to moderate kidney function loss.

### Statistical analysis

Power calculation was based on data from a previous similar study.^5^ The odds ratio (OR) among carriers was expected to exceed that for the overall African American population. With an alpha of 0.05 and 80% power, 255 participants with *APOL1* RVs were sufficient to detect an OR>2.8 for the association between smoking and CHD among carriers. Statistical analyses were conducted using R (version 4.4.1) software. Statistical significance was determined at *α* < 0.05 (2-sided). Variables were checked for missing values and skewness. Participants’ characteristics were summarized by smoking status. *P*-values and descriptive statistics are presented as mean ± SD or median (range) for continuous variables and as frequencies (%) for binary and categorical variables. Comparisons of distributions were performed by unpaired t-test for continuous variables or Pearson *χ*^2^ test for categorical variables.

To examine the relationship between smoking status and CHD, we used Firth’s logistic regression (‘logistf’ R package), categorizing smoking status as current vs nonsmokers (former and never smokers). Adjustments included age, sex, dyslipidemia, hypertension, diabetes, body mass index (BMI), and eGFR. Missing data were addressed using the “MICE” package, employing predictive mean matching (pmm) assuming data were missing at random. Due to significant missing data for alcohol and exercise (>30% of participants had missing values) these variables were omitted from the main model. Sensitivity analyses, including these variables among participants with complete data, showed no substantial impact on the magnitude of the association between smoking and CHD prevalence. To test for effect modification of the association between smoking and CHD history by *APOL1* genotype, an interaction term between the dichotomous smoking variable and *APOL1* genotype (yes/no) was included in multivariate models. In our secondary analysis, which was exploratory in nature, we examined the relationship between smoking and CHD separately among *APOL1* reference genotype subgroup, carriers of 1 risk allele, and carriers of 2 risk alleles (additive model). We also analyzed the association between current vs nonsmokers and prevalent MI stratified by *APOL1* genotype (dominant model).

In a sensitivity analysis, we assessed the association between smoking status (current, former, never) and CHD prevalence or MI, stratified by *APOL1* genotype, using a dominant model. In an additional exploratory analysis we performed a t-test to compare the average age of nonsmokers and current smokers with a history of CHD, overall and stratified by *APOL1* genotype, to assess potential differences in age of CHD onset.

## Results

### Participants’ characteristics

Participants’ characteristics are shown in Table 1; 72 (14%) were classified as current smokers, and 447 (86%) current nonsmokers, including 142 (27%) former, and 305 (59%) never smokers. The average age was 57.3 ± 14.0 years (18–88 years). Nonsmokers were older than current smokers (58.2 ± 14.2 years vs 52.9 ± 11.3 years), with slightly more women in the nonsmoker group (53%) and men in the current smoker group (55%). Median BMI was 27 kg/m^2^ (24–32 kg/m^2^), with no marked difference between nonsmokers and current smokers. There were also no significant differences between nonsmokers and current smokers concerning prevalence of diabetes, hypertension, or dyslipidemia. Nonsmokers included a higher proportion of physically active individuals (56% vs 13%) and fewer reported drinking >2 alcoholic drinks/week (42% vs 27%) when compared to current smokers. There were 146 participants with a history of prevalent CHD, of whom 29 (40%) were current smokers and 117 (26%) were nonsmokers. Of the 146 patients with CHD, 8 had insufficient information to determine MI status. Among the 138 CHD patients with available MI data, 82 (59%) had a history of MI. Additionally, 282 (54%) participants carried *APOL1* RVs (1 or 2 risk alleles), with 64 (12%) having 2 risk alleles and 218 (41%) having 1 risk allele. The *APOL1* G1 (rs73885319, *p* = .3126; rs60910145, *p* = .353) and the G2 variant (rs71785313, *p* = .3832) were consistent with Hardy-Weinberg equilibrium.

### Prevalent CHD and MI in current and nonsmokers differed by APOL1 genotypes

**Figure 1.**
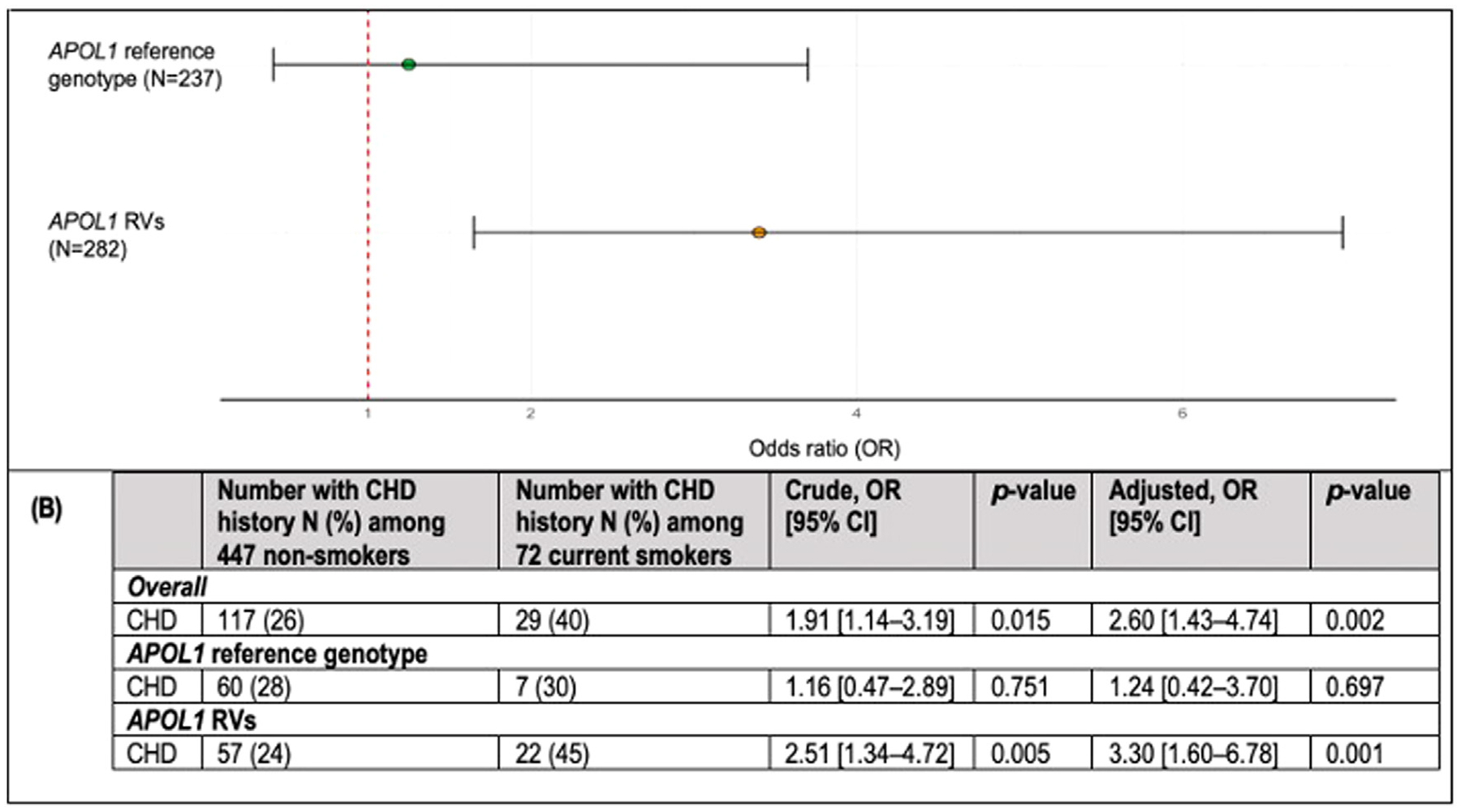
Prevalence odds ratio for CHD in current smokers compared to nonsmokers stratified by *APOL1* genotype. (A) Association between smoking and CHD overall and stratified by *APOL1* genotype groups in the cross-sectional study. ORs and 95% CIs were obtained from logistic regression with adjustments for age, sex, dyslipidemia, hypertension, diabetes, BMI, and eGFR using a dominant inheritance model for *APOL1* groups (at least 1 copy of the *APOL1* risk allele vs none) (B) Assessment of the number of CHD events in current and nonsmokers, overall and stratified by *APOL1* genotype groups. A detailed estimate of ORs, 95% CIs, and *p*-values on the association between smoking and CHD, both overall and within *APOL1* subgroups (dominant model), with and without adjustments for age, sex, dyslipidemia, hypertension, diabetes, BMI, and eGFR. Abbreviations: *APOL1*, apolipoprotein L1; BMI, body mass index; CHD, coronary heart disease; eGFR, estimated glomerular filtration rate; OR, odds ratio. *P*-values were obtained from logistic regression without and with adjustments for age, sex, dyslipidemia, hypertension, diabetes, BMI, and eGFR.

Carriers of the *APOL1* reference genotype showed a similar prevalence of CHD among current smokers and nonsmokers, 60 (28%) and 7 (30%), respectively, with a similar trend for MI. Among carriers of RVs, current smokers had a significantly higher prevalence of CHD compared to nonsmokers, (22 [45%] vs 57 [24%]) (Fig 1B) and had a significantly higher prevalence of MI compared to nonsmokers (14 [29%] vs 32 [14%]) (Table 2).

### APOL1 RVs associated with higher prevalence of CHD and MI among current smokers

The *p*-interaction suggested that the relationship between smoking and prevalent CHD may vary by *APOL1* genotype (*p* = .074), so we stratified the data by genotypes (Fig 1A,B).

Among 282 *APOL1* RV carriers, current smokers had 3.30 times the odds of having a history of CHD compared to nonsmokers (95% CI: 1.6, 6.8, *p* = .001). In contrast, current smoking was not significantly associated with a history of CHD among 237 individuals with the *APOL1* reference genotype (OR = 1.24; 95% CI: 0.4, 3.7, *p* = .697). A secondary analysis investigating the effect of genotype on the relationship between smoking status (current vs nonsmoker) and prevalent MI, was consistent with that for CHD (Table 2). The association between smoking and MI differed by genotype, with a significant association observed only among *APOL1* RV carriers (OR = 3.29; 95% CI: 1.5, 7.4).

### Additive effect of APOL1 risk alleles on CHD prevalence among current smokers

**Figure 2.**
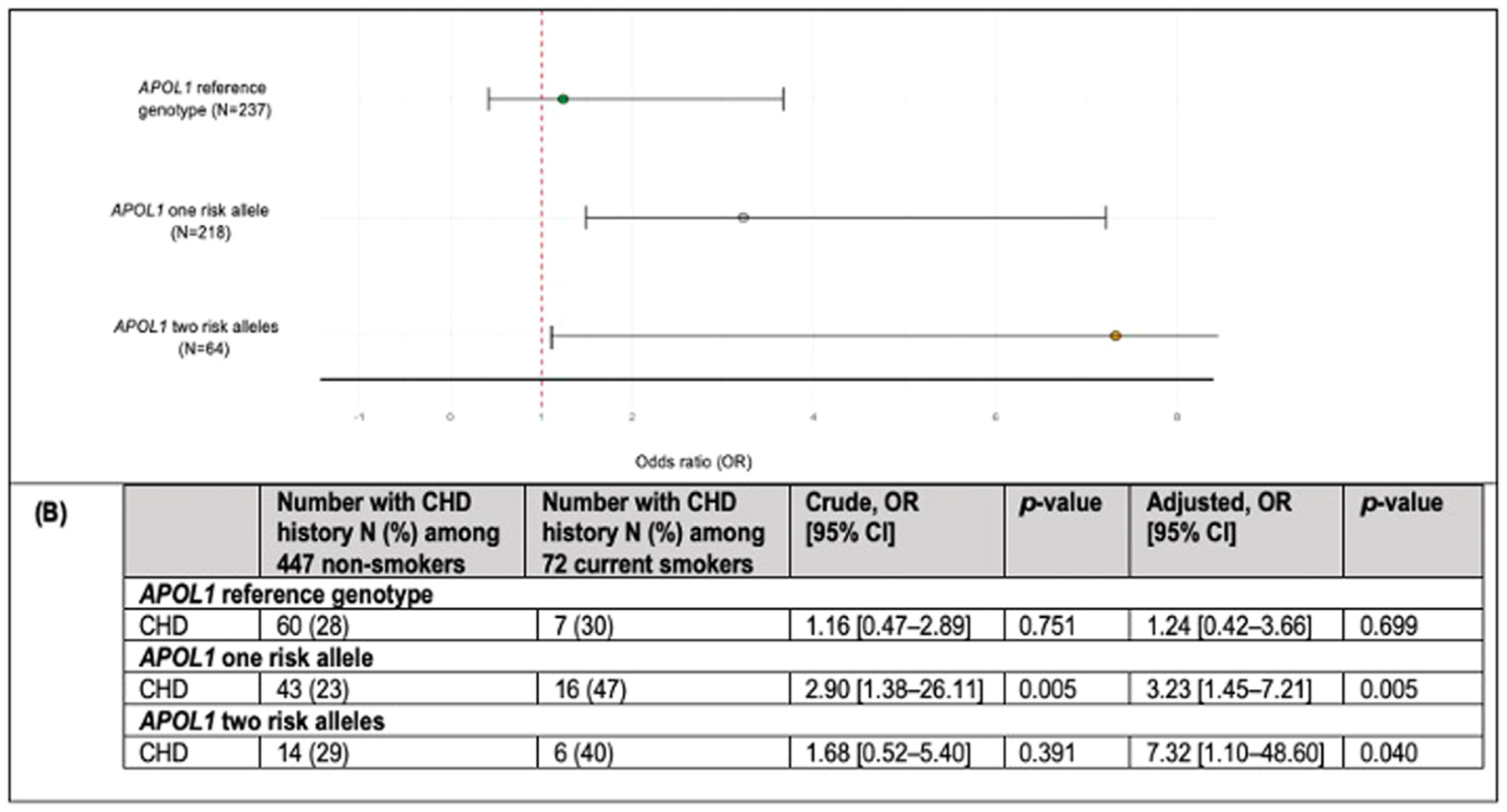
Prevalence odds ratio for CHD in current compared to nonsmokers stratified by copy number of *APOL1* alleles. (A) Association between smoking and CHD overall and stratified by 0, 1, and 2 copy number of *APOL1* alleles in the cross-sectional study. ORs and 95% CIs were obtained from logistic regression with adjustments for age, sex, dyslipidemia, hypertension, diabetes, BMI, and eGFR using an additive inheritance model for *APOL1* groups. (B) Assessment of the number of CHD events in current and nonsmokers, stratified by 0, 1, and 2 copy number of *APOL1* alleles. A detailed estimate of ORs, 95% CIs, and *p*-values on the association between smoking and CHD within *APOL1* subgroups (additive model) obtained from logistic regression, with and without adjustments for age, sex, dyslipidemia, hypertension, diabetes, BMI, and eGFR. Abbreviations: *APOL1*, apolipoprotein L1; BMI, body mass index; CHD, coronary heart disease; eGFR, estimated glomerular filtration rate; OR, odds ratio. *P*-values were obtained from logistic regression without and with adjustments for age, sex, dyslipidemia, hypertension, diabetes, BMI, and eGFR.

To assess whether the presence of 2 *APOL1* risk alleles had a stronger effect on the association between smoking and CHD than a single risk allele, we conducted secondary analyses in 3 subgroups (*APOL1* reference, 1 risk allele, and 2 risk alleles). The OR for CHD among current smokers compared to nonsmokers increased with the number of risk alleles (0, 1, or 2), with ORs (95% CI) of 1.24 (.4, 3.7), 3.23 (1.5, 7.2), and 7.32 (1.1, 48.6), respectively (Fig 2A,B).

### Smoking’s association with CHD and MI is independent of APOL1 genotype in former smokers

In the sensitivity analysis, current smokers among *APOL1* RV carriers had 3.63 times the odds of CHD compared to never smokers (95% CI: 1.7, 7.8, *p* = .001). The association between smoking and CHD was not significant for non-carriers (Table 3). Former smokers with RVs had 1.80 times the odds of having prevalent CHD compared to never smokers; this association did not reach statistical significance (95% CI: 0.9, 3.5, *p* = .090). A similar relationship was observed among non-carriers (OR = 2.01, 95% CI: 0.9, 4.3, *p* = .080). Among RV carriers, current smokers had 4.89 times the odds of MI history compared to never smokers (95% CI: 2.0, 12.0, *p* = .001, Table 4). The association was significant for former vs never smokers (OR = 2.66, 95% CI: 1.2, 5.7, *p* = .020). Among non-carriers, the relationship between past smoking and MI was significant (OR = 2.40, 95% CI: 1.0, 5.5, *p* = .043), whereas this was not observed for current smoking and MI (OR = 1.91, 95% CI: 0.6, 6.6, *p* = .331).

### Smokers with APOL1 risk variants may have earlier onset of CHD

Among participants with CHD history, current smokers were younger than nonsmokers; average age 57 years compared to 63 years (*p* = .018). Within the *APOL1* reference group, among those with prevalent CHD, the average age was similar between current smokers and nonsmokers (63 vs 63 years). For carriers of RVs, current smokers with prevalent CHD were significantly younger than nonsmokers; average age 55.5 years compared to 62 years (*p* = .017) (Table S1).

## Discussion

In this well-characterized cohort of African American adults, *APOL1* genotype status modified the association between current cigarette smoking and the odds of CHD, including MI. Among individuals with *APOL1* RVs, current smokers had 3.3 times the odds of CHD compared to nonsmokers, after adjusting for traditional risk factors. The effect was strongest in carriers of 2 risk alleles (OR = 7.3) and substantial for those with 1 risk allele (OR = 3.2), implying additive effect of these alleles. Among non-carriers, smokers had 1.2 times the odds of CHD, but this was not statistically significant. Given that approximately half of African Americans carry an *APOL1* risk allele, these findings highlight a potential interplay between genetic susceptibility and smoking that could contribute to the elevated rates of tobacco-related CHD in this population.

The concept of a *second hit* in the development of *APOL1* RV-associated diseases has been suggested in many studies.^18–20,30,31^ *APOL1* RVs are strongly linked to the excess burden of kidney disease among African Americans.^10,13–15^ In a mouse study, kidney disease development in carriers of *APOL1* RVs depended not only on the presence of G1 and G2 variants but also on their expression levels.^18^ In animal models and cell cultures, *APOL1*-induced toxicity was influenced by both the presence of RVs and expression levels, with higher *APOL1* mRNA or protein expression associated with increased toxicity.^18,32^ Moreover, human kidney glomerular samples show that elevated *APOL1* transcript levels are linked to kidney disease and correlate with kidney function.^18,32^ Inflammatory cytokines, such as IFN-*γ* and TNF, enhance *APOL1* gene expression in human umbilical vein endothelial cells (HUVECs),^11,33^ and even more so in human coronary artery endothelial cells (HCAECs), compared to podocytes.^9^ Studies investigating the relationship between *APOL1* RVs and CHD have yielded mixed results.^17,21,34–37^ Variations in cohort characteristics and the presence of additional risk factors, such as proinflammatory conditions, may alter the influence of *APOL1* RVs on CHD outcomes. As reported by Gutierrez et al.,^16^ hazard ratios (HRs) for the association between *APOL1* RVs and CHD under recessive and dominant models in nondiabetics indicate that a single risk allele does not increase CHD risk. Among diabetics, HR increased in the dominant model.^16^ Although these results were not statistically significant, the trend suggests that even 1 *APOL1* risk allele may elevate CHD risk, potentially due to diabetes-related inflammation. This implies that an additional insult is particularly relevant for disease development in carriers of 1 risk allele; carrying 2 may be sufficient to cause disease and the presence of additional insults may further exacerbate the condition. In our study, we observed a significant association between smoking and prevalent CHD among carriers of *APOL1* RVs, while the association did not reach significance among non-carriers. The effect of smoking was strongest in carriers of 2 risk alleles (OR = 7.3), followed by carriers of 1 (OR = 3.2), and less pronounced in non-carriers (OR = 1.2). It is important to note that the subgroup of smokers with 2 *APOL1* risk alleles was small, resulting in a wide 95% CI and limiting the precision of the effect size estimate. Thus, these findings should be interpreted with caution. Still, the results suggest that the effect is greater in individuals with 2 risk alleles compared to those with only 1, providing valuable insights that warrant further investigation in larger, more statistically powered studies. Overall, these results support the hypothesis that a *second hit* is important in *APOL1* RV-associated CHD development, with smoking, characterized by systemic inflammation, potentially acting as this *second hit* in RV carriers, leading to increased vulnerability to CHD possibly consistent with an additive inheritance model.

Pathologies associated with *APOL1* RVs are linked to endothelial cell dysfunction, with several studies highlighting the critical role of these cells in the disease development.^11,13–15^ Endothelial cells in vessels form the inner lining of the circulatory system with their dysfunction often preceding the onset of cardiovascular disease.^38^ Recently, *APOL1* RV carriers were found to have increased levels of adhesion molecules (vascular cell adhesion molecule-1 [VCAM-1] and intercellular adhesion molecule-1 [ICAM-1]) and a proinflammatory phenotype in kidney endothelial cells leading to their dysfunction.^13^ Endothelial cell dysfunction could lead to atherosclerosis and coronary plaque enlargement by increasing monocyte recruitment into vessel walls, their differentiation into macrophages, and greater low-density lipoprotein uptake. In transgenic mice, *APOL1* RVs were found to promote cholesterol accumulation in macrophages by downregulating key transporters involved in reverse cholesterol transport, leading to foam cell formation and growth of the necrotic core and plaque.^39^ Cornelissen et al.^21^ found *APOL1* RVs increased the risk of thrombotic coronary death due to plaque rupture.^21^ Specifically, individuals carrying risk alleles showed higher APOL1 protein accumulation within coronary plaques and larger necrotic core areas compared to non-carriers. In our study, the association between smoking and increased odds of prevalent CHD, including its most severe subtype, MI, was found to be strong and significant among carriers of *APOL1* RVs, but nonsignificant among non-carriers. *APOL1* RVs may contribute to the development of tobacco-related CHD by exacerbating the atherosclerotic processes in carriers. Indeed, tobacco smoking is known to elevate inflammatory markers in the circulation,^22,23^ and because endothelial cells are directly exposed to blood, RV carriers may experience heightened endothelial dysfunction and accelerated atherosclerosis, leading to increased tobacco-related CHD risk. Further experimental studies are required to confirm this hypothesis.

To isolate the effect of current smoking on CHD in the context of *APOL1* RVs, we classified current smokers using plasma biomarker data, allowing for a detailed assessment of current smoking status. However, the inclusion of former smokers in the nonsmoker group may have attenuated the observed association, particularly if residual risk persisted among recent quitters. This challenge arises from the self-reported nature of former smoking status, which lacked information on cessation timing. The prospective Jackson Heart Study, followed a large cohort of African Americans. CHD risk was significantly higher in current smokers (HR = 2.1) compared to never smokers and elevated for former smokers (HR = 1.4). In a sensitivity analysis we compared former and current smokers. The ORs for CHD were 2.0 for former and 3.3 for current smokers, highlighting the substantial adverse impact of continued smoking and the lower but still notable risk among former smokers. While the association between current smoking and CHD differed by *APOL1* genotype, with a strong positive association observed only in carriers, the association between former smoking and CHD did not vary by the genotype. This suggests that, though smoking has lasting effects on cardiovascular health, smoking cessation remains critically important, particularly for carriers of *APOL1* RV. As the duration of cessation significantly influences CHD risk—with longer abstinence consistently associated with greater risk reduction—future prospective studies with detailed data on historical smoking exposure, including the timing and duration of cessation, are warranted to understand better the potential interaction between past smoking behavior, *APOL1* RVs, and CHD risk.

Among smokers, most CHD cases across all age groups, regardless of sex, can be attributed to smoking.^24,40^ However, compared to never smokers, the relative risk of CHD among current smokers decreases with age.^40^ In a pooled data set of 192,067 women, the HR for CHD among current smokers aged 40 to 49 was 8.5 (95% CI: 5.0, 14), while for those over 70, it was 3.1 (95% CI: 2.0, 4.9).^40^ This suggests that either people susceptible to the adverse impact of smoking develop CHD earlier and/or die, or that the presence of other factors contribute more significantly to CHD risk as people age, reducing the smoking’s relative impact over time. Nevertheless, this implies a younger average age of CHD onset among smokers. Our findings align with this pattern, as smokers with prevalent CHD were, on average, younger than nonsmokers, particularly within the *APOL1* RV group highlighting smoking’s pronounced impact in RV carriers. In contrast, no significant age difference was observed between smokers and nonsmokers among non-carriers, possibly reflecting a smaller effect size or limited sample size. Our study design makes it difficult to determine whether the earlier CHD onset in RV carriers who smoke is a true phenomenon or influenced by survival bias. Future studies should use longitudinal data to accurately capture CHD onset in smokers with *APOL1* RV.

Our study has several limitations. First, the population was limited to participants who self-identified as African Americans, which restricts the generalizability of our findings to other racial or ethnic groups. However, this focus is justified, as *APOL1* RVs are almost exclusively found only in individuals of African descent. Second, due to the cross-sectional observational nature of our study, we are unable to establish causality between the variables examined. Further, it is possible that the associations we observed were in part due to associations of the exposures on the duration of CHD, since we were limited to prevalent CHD events. Additionally, we cannot entirely rule out residual confounding, such as socioeconomic status. Recognizing socioeconomic differences between racial groups^41^ and aiming to minimize the potential confounding effect of socioeconomic status—given that socioeconomic status may contribute to increased systemic inflammation^42^—we included only individuals who self-identified as African American in the study. Third, the lack of a significant association between smoking and CHD for non-carriers may reflect insufficient study power to detect a smaller effect size, and larger studies may be needed. Fourth, in the primary analysis, we applied a dominant model. While this approach does not account for a potential additive effect, emerging evidence suggests that even a single risk allele may increase susceptibility to CHD in smokers. A secondary analysis examined the association between smoking and CHD separately for non-carriers, carriers of 1 risk allele, and carriers of 2 risk alleles. This provided a more nuanced understanding of how the number of risk alleles influences CHD risk in the context of smoking.

In conclusion, this study is the first to examine the role of *APOL1* RVs in CHD among African American smokers, utilizing plasma smoking biomarkers to validate self-reported smoking status. With over 500 African American participants, our findings highlight the potential role of genetic susceptibility, specifically the presence of *APOL1* RVs, in modulating the impact of tobacco smoking on the prevalence of CHD, including MI. We observed a strong association between smoking and a history of CHD among carriers of RVs, with a dose-dependent increase in the odds of CHD corresponding to the number of risk alleles. While highlighting the overall harm of tobacco smoking, this study underscores its particularly severe adverse impact on individuals with *APOL1* RVs, which are highly prevalent among African Americans. These findings have significant public health implications, emphasizing the need for increased awareness and tailored smoking cessation programs within this historically marginalized community. They underscore the importance of personalized healthcare strategies that integrate genetic factors into risk assessments and offer insights into molecular targets for developing targeted interventions.

## Supplementary Material

Supplementary Material

Supplementary material associated with this article can be found, in the online version, at doi:10.1016/j.jacl.2025.04.189.

## Figures and Tables

**Table 1.** **Participants’ characteristics**.

	All (N = 519)	Nonsmoker (N = 447)	Current smoker (N = 72)	*p*-value
Age (years)				
Mean ± SD	57.5 ± 14.0	58.2 ± 14.2	52.9 ± 11.3	<.001
Median (IQR)	58 (50, 68)	59 (51, 69)	53 (45, 60)	
Sex (Male)				
N (%)	250 (48)	208 (47)	42 (58)	.083
BMI (kg/m^2^)				
Median (IQR)	27 (24, 32)	27 (24, 31)	27 (23, 31)	.192
Missing N (%)	37 (7)	36 (8)	1 (1)	
Hypertension, N (%)	282 (55)	239 (54)	43 (62)	.266
Missing, N (%)	10 (2)	7(1)	3 (4)	
Diabetes (Yes)				
N (%)	139 (27)	123 (28)	16 (23)	.413
Missing, N (%)	9 (2)	8 (2)	1 (1)	
Dyslipidemia (Yes)				
N (%)	227 (50)	194 (50)	33 (49)	1
Missing, N (%)	62 (12)	57 (13)	5 (7)	
Exercise (Yes)				
N (%)	182 (53)	155 (56)	27 (42)	.068
Missing, N (%)	177 (34)	169 (38)	8 (11)	
Alcohol intake (Yes)				
N (%)	56 (16)	39 (13)	17 (27)	.015
Missing, N (%)	163 (31)	155 (34)	3 (4)	
TC (mg/dL)	202.7 ± 77.9	201.7 ± 78.2	208.9 ± 76.3	.457
LDL-C (mg/dL)	117.8 ± 51.3	117.7 ± 50.4	118.8 ± 57.3	.874
Missing	34	28	6	
HDL-C (mg/dL)	57.7 ± 20.3	57.9 ± 19.7	56.6 ± 23.9	.666
Missing	24	21	3	
TG (mg/dL)	147.1 ± 307.8	143.3 ± 323.7	170.9 ± 180.3	.294
Missing	1	1	0	
Creatinine (mg/dL)				
Mean ± SD	1.52 ± 1.0	1.49 ± 0.8	1.80 ± 1.7	.200
Missing, N (%)	28 (5)	17 (4)	10 (14)	
eGFR<60 (mL/min/1.73m^2^)	315 (61)	264 (63)	41 (59)	.160
Missing, N (%)	28 (5)	25 (6)	3 (4)	
CHD				
N (%)	146 (28)	117 (26)	29 (40)	.020
MI				
N (%)	82 (16)	64 (15)	18 (26)	.028
Missing, N (%)	8 (2)	6 (1)	2 (3)	
*APOL1* RVs (Yes)	282 (54)	233 (52)	49 (69)	.017

Abbreviations: *APOL1*, apolipoprotein L1; BMI, body mass index; CHD, coronary heart disease; CRP, C-reactive protein; eGFR, estimated glomerular filtration rate; HDL-C, high-density lipoprotein cholesterol; LDL-C, low-density lipoprotein cholesterol; MI, myocardial infarction; RVs, rare variants; TC, total cholesterol; TG, triglycerides.

Results are presented as means ± SD, or N (percentages).

*P*-values denote differences between smoking groups (*current* smoker vs *non*smoker).

Exercise (Yes) was defined as engaging in physical activity, including walking, for at least 30 minutes more than twice per week. Alcohol intake (Yes) was classified as consuming more than 2 alcoholic drinks per week.

**Table 2.** Prevalence odds ratio for MI in current compared to nonsmokers stratified by *APOL1* genotype.

	Number with MI history N (%) among 447 nonsmokers	Number with CHD history N (%) among 70 current smokers	Crude, OR (95% CI)	*p*-value	Adjusted, OR (95% CI)	*p*-value
*Overall*						
MI	64 (15)	18 (26)	2.06 (1.14–3.73)	.021	2.54 (1.32–4.91)	.007
*APOL1* Reference Genotype						
MI	32 (15)	4 (18)	1.34 (0.45–4.02)	.606	1.30 (0.40–4.25)	.676
*APOL1* RVs						
MI	32 (14)	14 (29)	2.57 (1.25–5.26)	.013	3.29 (1.47–7.35)	.005

Abbreviations: *APOL1*, apolipoprotein L1; BMI, body mass index; CHD, coronary heart disease; eGFR, estimated glomerular filtration rate; MI, myocardial infarction; OR, odds ratio; RVs, rare variants.

*P*-values were obtained from logistic regression without and with adjustments for age, sex, dyslipidemia, hypertension, diabetes, BMI, and eGFR.

**Table 3.** Prevalence odds ratio for CHD in current smokers, former smokers compared to never smokers stratified by *APOL1* genotype group.

	Cases N (%) among 305 never smokers	Cases N (%) among 142 former smokers	Cases N (%) among 72 current smokers	Former vs Never, Adjusted OR (95% CI)	*p*-value	Current vs Never, Adjusted OR (95% CI)	*p*-value
*Overall*							
CHD	54 (18)	63 (44)	29 (40)	1.99 (1.21–3.28)	.007	3.29 (1.76–6.13)	.012
*APOL1* reference genotype							
CHD	26 (18)	34 (50)	7 (30)	2.01 (0.94–4.31)	.080	1.64 (0.54–4.96)	.399
*APOL1* RVs							
CHD	28 (18)	29 (39)	22 (44)	1.79 (0.92–3.47)	.090	3.63 (1.68–7.84)	.001

Abbreviations: *APOL1*, apolipoprotein L1; BMI, body mass index; CHD, coronary heart disease; eGFR, estimated glomerular filtration rate; OR, odds ratio; RVs, rare variants.

*P*-values were obtained from logistic regression with adjustments for age, sex, dyslipidemia, hypertension, diabetes, BMI, and eGFR.

**Table 4.** Prevalence odds ratio for MI in current smokers, former smokers compared to never smokers stratified by *APOL1* genotype group

	Cases N (%) among 305 never smokers	Cases N (%) among 142 former smokers	Cases N (%) among 70 current smokers	Former vs Never, Adjusted, OR (95% CI)	*p*-value	Current vs Never, Adjusted, OR (95% CI)	*p*-value
*Overall*							
MI	24 (8)	40 (29)	18 (26)	2.66 (1.49–4.77)	.001	3.85 (1.88–7.89)	<.001
*APOL1* reference							
MI	12 (8)	20 (31)	4 (18)	2.40 (1.04–5.53)	.043	1.91 (0.55–6.62)	.331
*APOL1* RVs							
MI	12 (8)	20 (27)	14 (29)	2.59 (1.17–5.72)	.020	4.89 (2.00–11.97)	.001

Abbreviations: *APOL1*, apolipoprotein L1; BMI, body mass index; CHD, coronary heart disease; eGFR, estimated glomerular filtration rate; MI, myocardial infarction; OR, odds ratio; RVs, rare variants.

*P*-values were obtained from logistic regression with adjustments for age, sex, dyslipidemia, hypertension, diabetes, BMI, and eGFR.

## References

[1] Kyalwazi AN, Loccoh EC, Brewer LC, Disparities in cardiovascular mortality between Black and White adults in the United States, 1999 to 2019. Circulation. 2022;146(3):211–228. doi:10.1161/CIRCULATIONAHA.122.060199. 35861764 PMC9310198

[2] U.S. Department of Health and Human Services Office of Minority Health. (https://minorityhealth.hhs.gov/omh/browse.aspx?lvl=4&lvlid=19). Accessed September 13, 2024.

[3] Mensah GA. Cardiovascular diseases in African Americans: fostering community partnerships to stem the tide. Am J Kidney Dis. 2018;72(5 Suppl 1):S37–S42. doi:10.1053/j.ajkd.2018.06.026. 30343722 PMC6200348

[4] Nguyen-Grozavu FT, Pierce JP, Sakuma KK, Widening disparities in cigarette smoking by race/ethnicity across education level in the United States. Prev Med. 2020;139:106220. doi:10.1016/j.ypmed.2020.106220. 32693179 PMC7494609

[5] Oshunbade AA, Kassahun-Yimer W, Valle KA, Cigarette smoking, incident coronary heart disease, and coronary artery calcification in Black adults: the Jackson Heart Study. J Am Heart Assoc. 2021;10(7):e017320. doi:10.1161/JAHA.120.017320. 33754833 PMC8174312

[6] Limou S, Nelson GW, Kopp JB, Winkler CA. APOL1 kidney risk alleles: population genetics and disease associations. Adv Chronic Kidney Dis. 2014;21(5):426–433. doi:10.1053/j.ackd.2014.06.005. 25168832 PMC4157456

[7] Friedman DJ, Pollak MR. Apolipoprotein L1 and kidney disease in African Americans. Trends Endocrinol Metab. 2016;27(4):204–215. doi:10.1016/j.tem.2016.02.002. 26947522 PMC4811340

[8] Smith EE, Malik HS. The apolipoprotein L family of programmed cell death and immunity genes rapidly evolved in primates at discrete sites of host-pathogen interactions. Genome Res. 2009;19(5):850–858. doi:10.1101/gr.085647.108. 19299565 PMC2675973

[9] Nichols B, Jog P, Lee JH, Innate immunity pathways regulate the nephropathy gene apolipoprotein L1. Kidney Int. 2015;87(2):332–342. doi:10.1038/ki.2014.270. 25100047 PMC4312530

[10] Friedman DJ, Pollak MR. APOL1 Nephropathy: from genetics to clinical applications. Clin J Am Soc Nephrol. 2021;16(2):294–303. doi:10.2215/CJN.15161219. 32616495 PMC7863644

[11] Blazer A, Qian Y, Schlegel MP, APOL1 variant-expressing endothelial cells exhibit autophagic dysfunction and mitochondrial stress. Front Genet. 2022;13:769936. doi:10.3389/fgene.2022.769936. 36238153 PMC9551299

[12] Cooper A, Ilboudo H, Alibu VP, APOL1 renal risk variants have contrasting resistance and susceptibility associations with African trypanosomiasis. Elife. 2017;6. doi:10.7554/eLife.25461. PMC5495568 28537557

[13] Wu J, Ma Z, Raman A, APOL1 risk variants in individuals of African genetic ancestry drive endothelial cell defects that exacerbate sepsis. Immunity. 2021;54(11):2632–2649 e6. doi:10.1016/j.immuni.2021.10.004. 34715018 PMC9338439

[14] Bird L APOL1 variants contribute to racial disparity in sepsis. Nat Rev Immunol. 2021;21(12):759. doi:10.1038/s41577-021-00647-3. 34707254 PMC8548855

[15] Carracedo M, Ericson E, Agren R, APOL1 promotes endothelial cell activation beyond the glomerulus. iScience. 2023;26(6):106830. doi:10.1016/j.isci.2023.106830. 37250770 PMC10209455

[16] Gutierrez OM, Irvin MR, Chaudhary NS, APOL1 Nephropathy risk variants and incident cardiovascular disease events in community-dwelling Black adults. Circ Genom Precis Med. 2018;11(6):e002098. doi:10.1161/CIRCGEN.117.002098. 29899045 PMC6339526

[17] Bick AG, Akwo E, Robinson-Cohen C, Association of APOL1 risk alleles with cardiovascular disease in Blacks in the million veteran program. Circulation. 2019;140(12):1031–1040. doi:10.1161/CIRCULATIONAHA.118.036589. 31337231 PMC6754626

[18] Beckerman P, Bi-Karchin J, Park AS, Transgenic expression of human APOL1 risk variants in podocytes induces kidney disease in mice. Nat Med. 2017;23(4):429–438. doi:10.1038/nm.4287. 28218918 PMC5603285

[19] Rakic JM, Pullinger CR, Van Blarigan EL, APOL1 Risk variants associate with the prevalence of stroke in African American current and past smokers. J Am Heart Assoc. 2023;12(24):e030796. doi:10.1161/JAHA.123.030796. 38084718 PMC10863786

[20] Langefeld CD, Comeau ME, Ng MCY, Genome-wide association studies suggest that APOL1-environment interactions more likely trigger kidney disease in African Americans with nondiabetic nephropathy than strong APOL1-second gene interactions. Kidney Int. 2018;94(3):599–607. doi:10.1016/j.kint.2018.03.017. 29885931 PMC6109415

[21] Cornelissen A, Fuller DT, Fernandez R, APOL1 Genetic variants are associated with increased risk of coronary atherosclerotic plaque rupture in the Black population. Arterioscler Thromb Vasc Biol. 2021;41(7):2201–2214. doi:10.1161/ATVBAHA.120.315788. 34039022 PMC8651054

[22] Shiels MS, Katki HA, Freedman ND, Cigarette smoking and variations in systemic immune and inflammation markers. J Natl Cancer Inst. 2014;106(11). doi:10.1093/jnci/dju294. PMC4200029 25274579

[23] Arnson Y, Shoenfeld Y, Amital H. Effects of tobacco smoke on immunity, inflammation and autoimmunity. J Autoimmun. 2010;34(3):J258–J265. doi:10.1016/j.jaut.2009.12.003. 20042314

[24] McEvoy JW, Blaha MJ, DeFilippis AP, Cigarette smoking and cardiovascular events: role of inflammation and subclinical atherosclerosis from the MultiEthnic Study of Atherosclerosis. Arterioscler Thromb Vasc Biol. 2015;35(3):700–709. doi:10.1161/ATVBAHA.114.304562. 25573855 PMC4404404

[25] Juliar BA, Stanaway IB, Sano F, Interferon-gamma induces combined pyroptotic angiopathy and APOL1 expression in human kidney disease. Cell Rep. 2024;43(6):114310. doi:10.1016/j.celrep.2024.114310. 38838223 PMC11216883

[26] Bay Area Census. Bay Area Census: A region by the numbers. (http://www.bayareacensus.ca.gov/bayarea.htm). Accessed February 21, 2025.

[27] Guey LT, Pullinger CR, Ishida BY, Relation of increased prebeta-1 high-density lipoprotein levels to risk of coronary heart disease. Am J Cardiol. 2011;108(3):360–366. doi:10.1016/j.amjcard.2011.03.054. 21757044

[28] Benowitz NL, Bernert JT, Caraballo RS, Holiday DB, Wang J. Optimal serum cotinine levels for distinguishing cigarette smokers and nonsmokers within different racial/ethnic groups in the United States between 1999 and 2004. Am J Epidemiol. 2009;169(2):236–248. doi:10.1093/aje/kwn301. 19019851

[29] Pullinger CR, Hennessy LK, Chatterton JE, Familial liganddefective apolipoprotein B. Identification of a new mutation that decreases LDL receptor binding affinity. J Clin Invest. 1995;95(3):1225–1234. doi:10.1172/JCI117772. 7883971 PMC441461

[30] Kudose S, Batal I, Santoriello D, Kidney biopsy findings in patients with COVID-19. J Am Soc Nephrol. 2020;31(9):1959–1968. doi:10.1681/ASN.2020060802. 32680910 PMC7461665

[31] May RM, Cassol C, Hannoudi A, A multi-center retrospective cohort study defines the spectrum of kidney pathology in Coronavirus 2019 disease (COVID-19). Kidney Int. 2021;100(6):1303–1315. doi:10.1016/j.kint.2021.07.015. 34352311 PMC8328528

[32] Beckerman P, Susztak K. APOL1: the balance imposed by infection, selection, and kidney disease. Trends Mol Med. 2018;24(8):682–695. doi:10.1016/j.molmed.2018.05.008. 29886044 PMC6101980

[33] Zhaorigetu S, Wan G, Kaini R, Jiang Z, Hu CA. ApoL1, a BH3-only lipid-binding protein, induces autophagic cell death. Autophagy. 2008;4(8):1079–1082. doi:10.4161/auto.7066. 18927493 PMC2659410

[34] Ito K, Bick AG, Flannick J, Increased burden of cardiovascular disease in carriers of APOL1 genetic variants. Circ Res. 2014;114(5):845–850. doi:10.1161/CIRCRESAHA.114.302347. 24379297 PMC3982584

[35] Hughson MD, Hoy WE, Mott SA, Bertram JF, Winkler CA, Kopp JB. APOL1 Risk variants independently associated with early cardiovascular disease death. Kidney Int Rep. 2018;3(1):89–98. doi:10.1016/j.ekir.2017.08.007. 29340318 PMC5762961

[36] Chen TK, Appel LJ, Grams ME, APOL1 Risk variants and cardiovascular disease: results from the AASK (African American Study of Kidney Disease and Hypertension). Arterioscler Thromb Vasc Biol. 2017;37(9):1765–1769. doi:10.1161/ATVBAHA.117.309384. 28572159 PMC5570668

[37] Langefeld CD, Divers J, Pajewski NM, Apolipoprotein L1 gene variants associate with prevalent kidney but not prevalent cardiovascular disease in the systolic blood pressure intervention trial. Kidney Int. 2015;87(1):169–175. doi:10.1038/ki.2014.254. 25029429 PMC4281289

[38] Lee WH, Ong SG, Zhou Y, Modeling cardiovascular risks of E-cigarettes with Human-induced pluripotent stem cell-derived endothelial cells. J Am Coll Cardiol. 2019;73(21):2722–2737. doi:10.1016/j.jacc.2019.03.476. 31146818 PMC6637948

[39] Ryu JH, Ge M, Merscher S, APOL1 renal risk variants promote cholesterol accumulation in tissues and cultured macrophages from APOL1 transgenic mice. PLoS One. 2019;14(4):e0211559. doi:10.1371/journal.pone.0211559. 30998685 PMC6472726

[40] Tolstrup JS, Hvidtfeldt UA, Flachs EM, Smoking and risk of coronary heart disease in younger, middle-aged, and older adults. Am J Public Health. 2014;104(1):96–102. doi:10.2105/AJPH.2012.301091. 23763425 PMC3910023

[41] Williams DR, Priest N, Anderson NB. Understanding associations among race, socioeconomic status, and health: patterns and prospects. Health Psychol. 2016;35(4):407–411. doi:10.1037/hea0000242. 27018733 PMC4817358

[42] Muscatell KA, Brosso SN, Humphreys KL. Socioeconomic status and inflammation: a meta-analysis. Mol Psychiatry. 2020;25(9):2189–2199. doi:10.1038/s41380-018-02592. 31628416 PMC6814496

