# Supplementary material for: Increased prevalence of coronary heart disease among current smokers carrying APOL1 risk variants within the African American population
Source: J Clin Lipidol. Author manuscript; Available in PMC 2026 Feb 20. (PMC12922773; doi:10.1016/j.jacl.2025.04.189)
Supplement: Supplementary Material [file NIHMS2146196-supplement-Supplementary_Material.docx]

**Table S1.** Differences in age of 146 African American adults with history of CHD in current and *non*-smokers, stratified by *APOL1* genotype.

| **Group** | **Non-Smokers (N=117)** | **Current Smokers (N=29)** | ***p*-value** |
| --- | --- | --- | --- |
| ***Overall*** |  |  |  |
| Age, year (Mean ± SD) | 62.9 ± 13.5 | 57.4 ± 10.2 | 0.018 |
| Median [IQR] | 65 [55–71] | 59 [50–64] |  |
| ***APOL1* reference genotype** | | | |
| Age, year (Mean ± SD) | 63.5 ± 13.3 | 63.3 ± 9.8 | 0.962 |
| Median [IQR] | 66 [55–72] | 64 [61–67] |  |
| ***APOL1* RVs** | | | |
| Age, year (Mean ± SD) | 62.4 ± 13.8 | 55.5 ± 9.8 | 0.017 |
| Median [IQR] | 62 [55–71] | 56 [49–61] |  |

Abbreviations: *APOL1* (apolipoprotein L1), IQR (interquartile range). Results are presented as mean ± SD and median [IQR]. ^a^*p*-values were obtained from unpaired t*-*test and denote differences between smoking groups (current smoker vs non*-*smoker).
